# Effect of various chemical disinfectants on the mechanical properties of elastomeric chains

**DOI:** 10.6026/973206300211651

**Published:** 2025-06-30

**Authors:** Thasnim Jawhar Kallayil, Francis P.G, Joseph Sebastian, Biju Kalarickal, Arun Bosco Jerald, Terry Thomas Edathotty

**Affiliations:** 1 Department of Orthodontics & Dentofacial Orthopaedics, Mar Baselios Dental College, Kothamangalam, Ernakulam, Kerala, India

**Keywords:** Disinfection, elastomeric chains, colony forming units, ultimate tensile strength, isopropyl alcohol

## Abstract

Different cleaning procedures affected the mechanical characteristics of elastomeric chains. Hence, 255 samples, divided into 17 groups of 15 samples each, were tested for bacterial contamination and tensile strength using five disinfection procedures. Colonies on culture plates indicated contamination, while the universal testing machine determined ultimate tensile strength. The samples disinfected with 70% isopropyl alcohol (IPA) remained contaminated, as shown by colony growth, even though there was a statistically significant reduction in colonies compared to the controls. All samples examined after 1 and 10 cycles in the disinfectants had higher ultimate tensile strengths than controls.

## Background:

The term cross-infection refers to the inadvertent transfer of bacteria, viruses or other contaminants by direct or indirect contact between contaminated surfaces due to inadequate measures of disinfection/ sterilisation [1]. Any breach in the sterilisation or disinfection protocol can make the orthodontic clinic a potential source of cross-infection. This has increased the apprehensions of all stakeholders, especially in the backdrop of blood-borne infections like hepatitis B, HIV and the SARS-CoV-2 pandemic. The rise in prevalence of diseases caused by viruses such as Hepatitis B, C and HIV necessitates enhanced safety measurements to be followed to control the spread of infections, especially in the context of dental procedures [2, 3]. Prevention of cross-infection is important concerning professional, ethical and legal regards. Elastomer is a general term that encompasses all materials which, after substantial deformation, rapidly return to their original dimensions. Over the years, the quest for materials with superior properties led to the development of synthetic elastomers made of polyurethanes that promptly replaced natural rubber elastics. Lately, elastomers have become inevitable when it comes to ligation of arch-wires to brackets, as well as being used as the force-delivering element in space closures. The properties of elastomeric materials tend to get altered in the presence of moisture due to water sorption that facilitates slippage of molecules or polymer chains past one another, owing to the acceleration of force decay [4]. Most of these materials tend to lose 50% to 70% of their force in the first 24 hours. Researchers have attempted to determine the effect of alteration of the environment with regard to initial force delivery and force decay of elastomeric materials [5]. These alterations include changes in conditions that could exist within the oral cavity or those occurring during sterilisation or disinfection of elastomers before placement in the oral cavity. Elastomeric materials, before being used on the patient, go through various handling stages during processing, packaging and manipulation by the dental assistant or orthodontist. At each handling stage, the material is prone to contamination [6]. As per the CDC guidelines, such materials that contact oral mucosa have to be sterilised in an autoclave or the least has to be cold sterilised using high-level disinfectants [7]. The challenge before the orthodontist is to use a method that will provide effective disinfection without adversely affecting the properties of the material [8, 9]. The study was intended to assess the bacterial contamination of elastomeric chains (E-chains) as received from supplier, after disinfection with chemical agents such as 70% isopropyl alcohol (IPA) for 12 minutes, 2.4% Glutaraldehyde (GTA) for 12 minutes, 0.55% Orthopthalaldehyde (OPA) for 12 minutes and Formalin vapour in air tight chamber for 5 hours and to assess bacterial contamination of E-chains after autoclaving at 121°C, 15 lbs pressure for 15 minutes. Therefore, it is of interest to report the tensile strength of E-chains as received from the supplier, after one cycle and ten cycles of disinfection with the above-mentioned chemicals and to assess the tensile strength of E-chains after one cycle and ten cycles of autoclaving.

## Materials and Methods:

This *in vitro* experimental study was conducted in the Department of Orthodontics and the Department of Microbiology, Mar Baselios Dental College, Kothamangalam and the Central Institute of Petrochemicals Engineering and Technology, Kochi, using the convenient sampling technique.

The sample size estimation was done using the formula:

2*(Zα + Zβ)^2^ *(SD)^2^/d^2^

2*(1.96 + 0.842)^2^*(6)^2^/(6.14)^2^=15

Where, Zα = 1.96, Zβ = 0.842, SD = Standard deviation = 6, d = Difference in mean = 6.14 n=15 (1.96 + 0.842)^2^x(6)^2^÷(6.14)^2^ =15. Thus, the study consisted of 255 samples. Fresh, 6 cm long segments of short E-chains (3M Unitek) were used for this study. The samples that had visible defects were excluded. A total of 255 segments of elastomeric chains measuring 6 cm were kept exposed in the clinical environment on an instrument trolley for 1 week. After exposure, they were randomly allocated to 17 study groups and subjected to their respective assessments. To study the samples before disinfection, 15 samples were assessed for bacterial contamination by the CFU study. Another 15 strips were subjected to assessment of tensile strength. To study the bacterial contamination of E-chain disinfected using chemical agents (the agents studied are 2.4% GTA, 0.55% OPA and 70% IPA), samples from the respective study groups were immersed in the chemical disinfectants. After one disinfection cycle corresponding to 12 minutes, the respective samples were assessed for bacterial contamination by colony-forming units (CFU) study and tensile strength was assessed using the universal testing machine (UTM). To study the efficacy of autoclaving, samples were autoclaved (121°C, 15 lbs pressure and 15 minutes). After completion of 1 cycle, the respective samples were evaluated for bacterial contamination and for assessment of tensile strength in UTM. To study the effects of disinfection using formaldehyde tablets, samples were placed in an airtight chamber with formaldehyde tablets for 5 hours in a concentration of 10 tablets/dm^3^. After the holding period, respective samples were subjected to CFU study and tensile strength assessment. Another aspect of this study was to assess the effect of repeated cycles of disinfection/autoclaving on the tensile strength of the E-chain. For this, samples from the respective study groups were subjected to 10 cycles of disinfection/autoclaving as mentioned above at intervals of 24 hours. On completing 10 cycles, their tensile strength was assessed.

## Assessment of bacterial contamination by the CFU study:

The procedure was performed in the level 2 safety cabinet under strict aseptic conditions. All the samples except for those from groups 1 and 9 were washed thoroughly using sterile distilled water for 2 minutes so as to eliminate any residues of disinfectants. The samples were immersed in 10 ml Brain Heart Infusion (BHI) broth, vortexed and mixed for two minutes and incubated at 37°C for 48 hours. 10 µL from the inoculated broth was plated onto BHI agar with the help of an L-shaped spreader. Those which showed turbidity were diluted and 10 µL from the dilution was plated on the BHI agar with the help of a L-spreader and incubator at 37 °C for 48 hours and the number of CFU/ml was calculated using the formula: Total number of colonies x dilution factor / Volume of the culture plated (ml).

## Assessment of Tensile strength in UTM:

The samples were subjected to stretching in a UTM, clamped at either end of the crossheads. The stretching was performed at a speed of 500 mm /minute until the samples fractured. The force at breakage (tensile load at failure) was measured in Newtons, which corresponds to Ultimate Tensile Strength (UTS).

## Statistical analysis:

The statistical analysis was performed using SPSS software. The level of significance was set at 0.05. After statistical analysis, the data were segregated into individual tables as required by the study objectives and analysed using t-test and ANOVA.

## Results:

A total of 255 samples were randomly allocated to 17 study groups of 15 each. The samples were assessed for microbial contamination prior to disinfection and after one cycle of disinfection. The ultimate tensile strength was measured after one and 10 disinfection cycles. On gross evaluation, the number of colonies differed significantly among the various groups studied. All the BHI plates from group 1 had heavy growth and such plates were labelled as Too Many to Count. All the plates cultured from groups 5, 7, 9 and 11 had no growth at the end of 48 hours of incubation. For the samples disinfected using 70 % IPA (Group 3), the mean obtained was 1.18x109 CFU/ml. The difference in the observations between the study groups is deductive of a significant difference between groups 1 and 3 when compared to groups 5, 7 and 11, which had no growth of colonies (Table 1 - see PDF).

The distribution of data obtained after measurement of the UTS of the samples studied is provided in Table 2 (see PDF). The mean ultimate tensile strength (tensile load at failure) of the samples before disinfection (group 2 or control group) was 23.35±0.57 N (Table 2 - see PDF). For the samples disinfected using 70% IPA for one cycle (Group 4), the mean ultimate tensile strength obtained was 24.15±0.50 N. On comparing these values to those in Group 1 using an unpaired t-test, there is a statistically significant difference among the mean values obtained with a p-value of 0.001. For the samples subjected to 10 cycles of disinfection using 70% IPA (Group 13), the mean ultimate tensile strength obtained was 23.80±0.60 N. On comparing these values using an unpaired t-test to those in group 2, the p-value obtained was 0.066, suggestive of statistical insignificance. When samples disinfected using 70% IPA were compared between one cycle (Group 4) and 10 cycles (Group 13) using an unpaired t-test, there was a statistically significant difference (p=0.039). The comparison of mean values of samples disinfected using 2.4 % GTA for 1 cycle (Group 6) to those in the control group (Group 2) shows a statistically significant difference (p=0.002). When 10 disinfection cycles were carried out in samples (Group 14), the mean value measured was 24.15±0.29 N. On comparison with the control group (group 2), using an unpaired t-test, there was a statistically significant difference (p=0.000). The comparison between 1 cycle (group 6) and 10 cycles (group 14) showed a statistically insignificant difference (p-value 0.927) between the mean values (Table 3 - see PDF).

The samples disinfected using OPA for 1 cycle (Group 8) had mean UTS of 24.14±0.42 N. There is a statistically significant difference in the mean of samples between Group 8 and Group 2 (p=0.002). When samples were disinfected using OPA for 10 cycles (Group 15), the mean ultimate tensile strength obtained was 25.25±0.78 N. When the mean of group 15 was compared to that in the control group (Group 2), there was a statistically significant difference (p=0.000). When the mean values of samples between 1 and 10 disinfection cycles were compared, there was a statistically significant difference (p=0.0000). When samples in group 10 were autoclaved for 1 cycle (Group 10), the mean UTS measured was 27.14±0.66 N. On comparison with samples in group 10 with the control group, there was a statistically significant difference between them (p=0.000). When samples were subjected to 10 cycles of autoclaving (Group 16), the mean ultimate tensile strength obtained was 27.87±0.32 N. While comparing the mean values of group 16 to the control group, there was a statistically significant difference obtained (p=0.000). When the mean values of samples were compared between 1 cycle (group 10) and 10 cycles (Group 16) using an unpaired T-test, there was a statistically significant difference (p=0.004). The mean UTS measured for samples disinfected using formalin vapour for 1 cycle (Group 12) was 24.09±0.38 N. When the mean value of group 12 was compared to that of the control group (Group 2), there was a statistically significant difference with a p-value of 0.001. When samples subjected to 10 disinfection cycles using formalin vapour (group 17) were assessed for ultimate tensile strength, the mean value obtained was 25.09±0.58. When the mean value of group 17 was compared to that of the control group, there was a statistically significant difference between them with a p-value of 0.000. When samples disinfected using formal vapour for 1 cycle (Group 12) and 10 cycles (Group 17) were compared, there was a statistically significant observed between them with a p-value of 0.00 (Table 3 - see PDF). When the mean UTS of samples from groups 4, 6, 8, 10 and 12, disinfected using respective agents for one cycle, were compared using multivariate ANOVA, there was no statistically significant difference observed between them with a p-value of 0.00 (Table 4 - see PDF). Correspondingly, when the mean ultimate tensile strength of samples from groups 13, 14, 15, 16 and 17, disinfected using respective agents for 10 repeated cycles were compared using multivariate ANOVA, there was no significant difference in the mean values between all the groups analysed with p- value 0.00 (Table 4 - see PDF). The Post-Hoc multiple comparisons of UTS after one cycle and ten cycles of disinfection in all the agents studied was assessed using Tukey's HSD (Table 5 - see PDF).

## Discussion:

The results of the comparison of mean values of ultimate tensile strength obtained after 1 and 10 disinfection cycles using ANOVA showed statistically significant differences in both cycles. The results of multiple comparisons using Tukey's post-hoc analysis are suggestive of differences in the mean values that were statistically insignificant between the different methods of disinfection, except for autoclave, which showed a statistically significant difference when compared against all other disinfection methods used in our study (p<0.05). Purmal *et al.* [10] reported a study where they assessed the contamination of four different types of buccal tubes from three different manufacturers. The report of their study indicated the presence of contamination in some of the samples tested and confirmed the presence of viable aerobic bacterial species, which are potential causatives for nosocomial infections. These results are in concurrence with the results of this study, confirming the presence of contamination in the materials before clinical use. The authors recommended the need for sterilisation of buccal tubes before clinical use. Casaccia *et al.* [11] evaluated the presence of pathogenic microorganisms at the moment of unpacking of elastomeric chains from Ortho-Organisers Inc., 3M Unitek and Dental Morelli. The study report denies the presence of microbes in any of the samples tested at all incubation periods, which was contradictory to the results of our study. The results are suggestive of an inefficiency of 70% IPA in eliminating all the microbes. This may be attributed to the fact that alcohol, being an intermediate-level disinfectant, cannot kill all the microbes, including spores [7]. Pithon *et al.* [12] studied the effectiveness of various disinfectants in reducing microbial contamination by counting the number of colony-forming units and comparing them against a control group. 70% ethyl alcohol, autoclave, ultraviolet radiation, peracetic acid and 2% glutaraldehyde were the methods of disinfection used by them. The results of their study stated that all the methods used by them, except for the ultraviolet method, led to 100% decontamination of elastomeric chains with p<0.05. They suggested that disinfection using 70% ethyl alcohol for 1 minute is an effective method of disinfection, which was not in agreement with the present samples disinfected using 70% IPA and remained contaminated. This could probably be due to the difference in the composition of alcohol used by both studies, in spite of the concentration being 70%. Devi *et al.* [13] studied the efficacy of various disinfectants on the decontamination of dental impression surfaces. The results obtained by them indicated 2% Glutaraldehyde to be higher in efficacy in reducing the microflora when compared to other disinfectants used in their study. These findings are similar to those obtained in the present study. Evangelista *et al.* [14] noted temporal dependence and gradual deterioration of elastomers submerged in GTA solution. They claimed that the active chemical and water function by plasticising the elastic polymer, facilitating the slippage of polymeric chains relative to one another. The study conducted by Akamatsu *et al.* [15] evaluated the antimicrobial activity as well as material compatibility of OPA as a High-level Disinfectant. The results of their study showed that even the lowest concentration of OPA (0.25%) used by them was faster acting than GTA (3%) in terms of its antimicrobial effects against all of the 21 strains of microbes tested by them. The results led to the conclusion that OPA is an effective antimicrobial agent to be used as a first-replacement choice against GTA as a high-level disinfectant for endoscopes. The results of their study are in agreement with the findings from my study, which suggest 0.55% OPA to be an effective disinfectant for E-chains although formalin gas has been used as the gold standard for sterilising operating theatres in hospitals, providing high-level disinfection, its application in dentistry is scarce. The search through available literature did not reveal the use of formaldehyde vapour disinfection of orthodontic materials. In our study, Formalin vapour generated from formalin tablets placed in an air-tight chamber (Formalin chamber) has been studied to assess its effectiveness as a disinfectant for elastomeric chains. The protocol followed was based on recommendations by Suzuk *et al.* [16] and Schilling *et al.* [17]. The disinfection in the formalin chamber was carried out at room temperature with formaldehyde tablets added to a concentration of 10 tablets/ dm3. The results of this study prove formalin vapour to be an effective method of clinical disinfection of elastomeric chains, as suggested by the absence of growth of colonies.

Ardeshna *et al.* [18] studied the effectiveness of different sterilisation methods on clinical orthodontic materials, which included elastomeric chains. They used UV, dry heat, steam autoclave, ethyl alcohol and 2% glutaraldehyde to disinfect the materials. The results of their study were conclusive of effective removal of bacterial contamination using all five methods studied, which included autoclaving. The findings of their study are in agreement with the results of this study, suggesting that the autoclave is very efficient in decontamination. The results of Pithon *et al.* [12] are suggestive of 2% Glutaraldehyde being an effective disinfectant that does not cause deterioration of mechanical properties. The findings of their study are in agreement with the results obtained from this study using 2.4% glutaraldehyde. Sulaiman *et al.* [19] studied the effect of temperature and artificial saliva on the tensile force of orthodontic power chains. The tensile force was measured using a Correx meter force gauge with units of grams-force (gf) at the initial and final immersion. The temperatures used in their study were 4°C as obtained from the refrigerator, 23°C as obtained from storage at room temperature, 37°C as obtained from storage in an incubator and 55°C as obtained from heating with a hot plate. The results showed a significantly lower tensile force with P < 0.05 at different immersion temperatures. When the temperature, along with the effects of the immersion medium (artificial saliva solution and Aquadest), was studied, it resulted in a significant decline in tensile force at 23°C (p<0.05), whereas the results at 4°C, 37°C and 55°C were not significantly different. These findings are in favour of the claim that temperature affects the mechanical properties of elastomeric chains adversely. On the contrary, the results of the present study fail to show force degradation of elastomeric chains by subjecting them to autoclaving for 1 and 10 cycles. The parameters used in our study to assess the mechanical properties of E-chains were 'ultimate tensile strength', *i.e.*, the force level at which the elastomeric chain breaks. This is not a true measurement of the elasticity of the material. Therefore, the observation that autoclaving increased the UTS of elastomeric chains does not mean that the 'elasticity' of elastomeric chains improved as a result of autoclaving. Osorio *et al.* [20] examined the various cleaning procedures and their effect on the mechanical characteristics of orthodontic elastomeric ligatures (EL). Depending on the disinfection method used, 120 EL were allocated at random to one of six experimental groups: group 1 was not immersed in any disinfectant solution (control group); group 2 underwent immersion in 2% GTA; group 3 was immersed in 70% alcohol solution; group 4 was cleaned in an ultrasound washing (UW) machine by immersing them in a 0.5% enzyme detergent solution; group 5 underwent the UW process following an immersion in 2% GTA; Group 6 underwent immersion in 70% alcohol after completing the UW treatment. The only disinfectant that did not significantly modify the mechanical characteristics of orthodontic elastics was 2% GTA, which is regarded as a substitute for elastic disinfection before use. The results of their study are in agreement with the present study from the perspective that GTA could decontaminate the elastomeric chains. Baty *et al.* [21] stated that autoclaving at 121°C does not produce permanent deformation of elastomeric modules like observed after dry heating, but the modules tend to shrink, hence making them difficult to tie onto brackets which was in agreement with the present study findings, which could be the reason for the maximum increase in mean UTS obtained among all the methods studied here. The statistically significant decrease in the mean UTS values after 10 cycles of disinfection using 70% IPA can be a concern, as the clinical practice of wiping with alcohol is considered the most common chairside disinfection practice amongst orthodontists. Further studies have to be conducted to assess progression force deterioration with more than 10 disinfection cycles. The chemical agents used for disinfection in our study need to be evaluated further on human subjects to assess the biocompatibility so as to avoid any short and long-term adverse effects on patients and operating personnel.

## Conclusion:

The disinfection procedures in this investigation decontaminated elastomeric chains except for 70% IPA. After one cycle and 10 cycles of disinfection, all methods measured higher UTS than the control group, which was statistically significant. However, samples disinfected with 70% IPA for 10 cycles had statistically significantly lower mean UTS than samples treated with one cycle. The mean UTS for samples disinfected with 0.55% OPA, formalin vapour and an autoclave after 10 cycles was statistically greater than after one cycle.
